# Exercise Training to Improve Brain Health in Older People Living With HIV: Study Protocol for a Randomized Controlled Trial

**DOI:** 10.2196/41421

**Published:** 2023-03-21

**Authors:** Sarah Cooley, Brittany M Nelson, Alexander Rosenow, Elizabeth Westerhaus, W Todd Cade, Dominic N Reeds, Florin Vaida, Kevin E Yarasheski, Robert H Paul, Beau M Ances

**Affiliations:** 1 Department of Neurology School of Medicine Washington University in St. Louis Saint Louis, MO United States; 2 Doctor of Physical Therapy Division Duke University School of Medicine Durham, NC United States; 3 Department of Medicine and the Center for Human Nutrition Washington University in St. Louis Saint Louis, MO United States; 4 Division of Biostatistics and Bioinformatics School of Public Health University of California San Diego San Diego, CA United States; 5 Division of Endocrinology School of Medicine Washington University in Saint Louis Saint Louis, MO United States; 6 Department of Psychology University of Missouri St. Louis Saint Louis, MO United States; 7 Department of Radiology School of Medicine Washington University in St. Louis Saint Louis, MO United States

**Keywords:** cardiorespiratory fitness, cognition, exercise, HIV, magnetic resonance imaging, resistance training

## Abstract

**Background:**

With the advent of antiretrovirals, people living with HIV are living near-normal lifespans. However, people living with HIV are at greater risk of experiencing cognitive impairment and reduced brain integrity despite well-controlled viremia. A robust literature supports exercise interventions as a method of improving cognition and structural brain integrity in older individuals without HIV. The effects of exercise on cardiometabolic, neurocognitive, and neural structures in middle-aged to older people living with HIV are less well known, with few prospective studies examining these measures.

**Objective:**

This prospective randomized clinical trial will examine the effects of a 6-month exercise training intervention compared to a 6-month stretching intervention (control) on cardiorespiratory fitness, physical function and strength, cognition, and neuroimaging measures of brain volumes and cerebral blood flow in people living with HIV.

**Methods:**

Sedentary middle-aged to older people living with HIV (ages≥40; n=150) with undetectable HIV viral load (<20 copies/mL) will be enrolled in the study. At the baseline and final visit, fasting plasma lipid, insulin, glucose, and brain neurotrophic factor concentrations; cardiorespiratory fitness; cognitive performance; brain volumes; and cerebral blood flow via a magnetic resonance imaging scan will be measured. Participants will be randomized in a 2:1 ratio to either the exercise or control stretching intervention. All participants will complete their assigned programs at a community fitness center 3 times a week for 6 months. A professional fitness trainer will provide personal training guidance at all sessions for individuals enrolled in both arms. Individuals randomized to the exercise intervention will perform endurance and strength training exercises, while those randomized to the control intervention will perform stretches to increase flexibility. A midpoint visit (at 3 months) will assess cognitive performance, and at the end point visit, subjects will undergo cardiorespiratory fitness and cognition testing, and a magnetic resonance imaging scan. Physical activity throughout the duration of the trial will be recorded using an actigraph.

**Results:**

Recruitment and data collection are complete as of December 2020. Data processing, cleaning, and organization are complete as of December 2021. Data analysis began in January 2022, with the publication of study results for primary aims 1 and 2 expected by early 2023.

**Conclusions:**

This study will investigate the effects of a 6-month aerobic and resistance exercise training intervention to improve cardiometabolic risk factors, cognitive performance, cerebral structure, and blood flow in sedentary people living with HIV. Results will inform clinicians and patients of the potential benefits of a structured aerobic exercise training program on the cognitive, functional, and cardiometabolic health status of older people living with HIV. Assessment of compliance will inform the development and implementation of future exercise programs for people living with HIV.

**Trial Registration:**

ClinicalTrials.gov NCT02663934; https://clinicaltrials.gov/ct2/show/NCT02663934

**International Registered Report Identifier (IRRID):**

DERR1-10.2196/41421

## Introduction

### Overview

In the era of combination antiretroviral therapy (cART), people living with HIV are living near-normal lifespans [1]. Despite this, cognitive impairment is still prevalent in people living with HIV, even in those with well-controlled viremia [2]. Current adjunctive therapies to cART, such as cognitive training or other medications designed to treat comorbid conditions (ie, anticholinergics and lithium), have not demonstrated significant benefit in improving cognition and quality of life in people living with HIV. Consequently, there is a critical need for the development of adjunctive therapies that improve cognition in people living with HIV.

The effects of aerobic and resistance exercise (EXS) training interventions on physical and brain health have been well documented in people without HIV. These interventions are especially important in older adults, as older individuals tend to exhibit more sedentary lives than younger individuals [3]. Improved physical functioning and physical fitness have been reported in older people without HIV who completed exercise intervention programs [4-7]. Older adults have also demonstrated improvements in cardiorespiratory health (ie, VO_2_ maximum [VO_2__max_], the maximum rate of oxygen consumption during exercise) after completing an exercise program [8]. Reviews of the extant literature reveal that exercise intervention programs improve cognitive performance or slow cognitive decline, specifically in domains such as attention, processing speed, executive function, and memory [9-12] in both clinical populations (mild cognitive impairment and Alzheimer’s disease) and otherwise healthy older adults. Neuroimaging indices of brain integrity also improve in response to engagement in structured exercise interventions in older adults. Specifically, increased brain volumes [13,14], changes in cerebral blood flow (CBF) [15-17], and improved brain network connectivity [16,18,19] have been observed in individuals completing exercise interventions. These results are particularly evident in the frontal and temporal regions (including the hippocampus), each of which is critical for executive function, learning, and memory.

Research into the efficacy of an exercise intervention in improving physical and brain health in people living with HIV is less established. Longitudinal intervention studies have primarily focused on physical health outcomes, with evidence of improved blood pressures (BPs), cardiorespiratory fitness (VO_2 max_), inflammatory markers, physical strength, and body composition in people living with HIV following completion of an EXS training program or other activity programs, such as yoga [20-25]. The relationship between exercise and cognitive outcomes in people living with HIV has primarily been assessed using association studies and self-reported levels of physical activity. The effects of self-reported exercise or physical activity on cognitive function in the setting of HIV have produced conflicting results, with both positive [26-30] and null findings [31] reported. Because these studies did not include an intervention component, a causal effect of exercise on cognition was not able to be established.

Few studies have used neuroimaging to examine the effects of exercise on structural brain integrity in people living with HIV. While one cross-sectional study reported larger putamen volumes in a group of physically active people living with HIV compared to sedentary people living with HIV [30], to our knowledge, there have been no prospective studies examining neuroimaging markers of brain integrity before and after completion of an EXS training intervention in older people living with HIV. Therefore, the purpose of this clinical trial is to examine the effects of a 6-month EXS training intervention (vs a stretching control group) on cardiorespiratory and physical function, plasma markers of cardiometabolic risk and neurotrophic peptides, cognitive performance, and neuroimaging metrics in a sample of sedentary older people living with HIV who are on a stable cART regimen and virologically well controlled.

### Aims

The primary objective of this study is to compare the effects of supervised EXS training versus a social-interaction stretching (SIS) group on brain health in people living with HIV aged ≥40 years.

#### Aim 1

The aim is to examine the effects of exercise training on cognitive function in people living with HIV aged ≥40 years.

##### Hypothesis 1a

A 26-week EXS program will enhance cognitive function (domain-specific executive function, attention, and memory) more than an SIS program in physically inactive people living with HIV.

##### Hypothesis 1b

Greater improvements in cardiorespiratory fitness and physical function (daily physical activity, muscle strength, and muscle mass) in the EXS group will be associated with better cognitive performance.

#### Aim 2

The aim is to examine the effects of exercise training on brain structural and functional measures in people living with HIV aged ≥40 years.

##### Hypothesis 2a

A 26-week EXS program will associate with improved brain structural and functional measures (CBF and brain volumetrics) more than an SIS program in physically inactive people living with HIV.

##### Hypothesis 2b

Greater changes in physical function (daily physical activity, cardiorespiratory capacity, muscle strength, and muscle mass) in the EXS group will be associated with increases in CBF and larger brain volumetrics.

##### Hypothesis 2c

Improved neuroimaging changes will be associated with enhanced cognition in people living with HIV.

##### Exploratory Hypothesis

Improvements in brain integrity due to EXS will be accompanied by reduced systemic inflammation and improved glucose regulation.

## Methods

### Participants

All study visits are conducted at Washington University in St. Louis. Participants are recruited from local clinic referrals, flyers posted in pharmacies, food pantries, and advocacy groups in addition to word of mouth. Our team has also met with caseworkers and physicians to introduce them to this study.

Inclusion criteria are as follows: age 40-80 years, sedentary lifestyle (defined as structured exercise for less than 2 hours per week for the last 6 months), known HIV infection, on a stable cART regimen with an undetectable plasma HIV RNA (<50 copies/mL) for at least 12 months prior to enrollment, able to receive a magnetic resonance imaging (MRI) scan, and able to attend 3 EXS or SIS sessions a week over 6 months. It should be noted that the study inclusion criteria originally only enrolled older people living with HIV older than 50 years. However, due to difficulty in enrolling this population, the study opened enrollment to people living with HIV older than 40 years during the final year of enrollment.

Exclusion criteria are as follows: history of serious cardiovascular or cerebrovascular disorder (ie, stroke and uncontrolled hypertension), pulmonary disease, or significant neurological disorders; substance use disorder within the last 6 months; pregnant or breastfeeding; currently prescribed anticoagulants or medications known to affect glucose and lipid metabolism (other than statin medications); or any other medical condition that, in the opinion of the principal investigator, makes the patient unsuitable for inclusion in the study (eg, severe psychiatric illness).

### Study Visits

#### Baseline Visit 1

##### Cardiovascular Visit

After obtaining informed consent from each volunteer and after 10 minutes of rest, BP, heart rate (HR), temperature, waist circumference (mid-way between right costal margin and anterior superior iliac crest), height (by using a stadiometer), weight, and fasting blood glucose level (Accucheck) are collected. Female participants are administered a urine pregnancy test (McKesson Consult Diagnostics, manufacturer #5000) before testing begins.

Whole-body dual-energy x-ray absorptiometry (DXA) scan is performed on a GE-Healthcare Lunar I scanner. DXA outcome variables, quantified through the use of Encore Software (version 16), include BMI, total mass, lean mass, fat mass, android and gynoid mass, visceral adipose tissue mass, and fat tissue mass for each arm, leg, and the trunk.

Once the DXA is completed, an intravenous tube is placed in the participant’s arm for the blood draw (see Bloodwork section below). Once blood is collected, an electrocardiogram machine (Case V6.73) is obtained. The study physician then conducts a medical history and physical examination.

After the electrocardiogram, the participant completes a graded maximal exercise test on a recumbent cycle ergometer with concurrent indirect calorimetry (ParvoMedics True One System) with a 2-way valve and mouthpiece (Hans Rudolph Inc) in order to measure VO_2_. Test workload is initiated at 20 W. Once exercise starts, the participant is asked at 1-minute intervals to rate their perceived exertion on a scale of 6-20, and the resistance is increased by 10-20 W every minute until volitional exhaustion. HR is recorded once every minute, while BPs are collected every 2 minutes. After the session, the participant is monitored for 5 minutes until BP reaches baseline value. Maximal VO_2 max_ (mL O_2_/kg/min) is recorded as the highest VO_2_ reached during the test. Exercise tests are considered maximal if the participant reaches ≥85% projected peak HR (220 – age) or respiratory exchange ratio ≥1.1 [32].

After testing, an actigraph (ActiGraph GT3X+) is attached to the nondominant wrist of each subject to record daily physical activity. This actigraph is initialized using the ActiLife v6.13.4 program and set to start recording 1 hour after the end of the study visit with a sampling rate of 100 Hz. The actigraph is worn continuously for 1 week and is collected at baseline visit 2. Once returned, actigraph data are downloaded and integrated into a 60-second epoch-length format to be analyzed using ActiLife. Average time spent sedentary, lightly active, moderately active, vigorously active, and in moderate-to-vigorous physical activity are the primary outcome variables generated by the ActiLife scoring program.

##### Bloodwork

Fasting (12 hours) samples are collected during the cardiovascular visit and sent to the Core Laboratory for Clinical Sciences, Barnes Jewish Hospital, Washington University in St. Louis; Quest Diagnostics; or Temple University for processing. The following measures are reported from these samples: comprehensive metabolic panel and lipid panel, C-reactive protein, complete blood count, hemoglobin A1C, insulin and c-peptide, insulin-like growth factor 1, CD4 T-cell count, CD8 T-cell count, HIV RNA viral load, interleukin-6, C-X-C motif chemokine ligand 10, vascular endothelial growth factor, apolipoprotein E, leptin, neopterin, adiponectin, as well as archived plasma and serum. For serum, blood is allowed to clot for 30 minutes and then centrifuged at 2000×*g* at 4 °C for 10-15 minutes before aliquoting and storing in 80 °C freezer. For plasma, samples are centrifuged at 1500×*g* at 4 °C for 10 minutes before aliquoting and storing in 80 °C freezer.

#### Baseline Visit 2

##### MRI Scan Visit

One to 2 weeks after the initial visit, MRI scanning and neuropsychological (NP) evaluation are performed. An MRI safety form is completed before the scan to ensure there are no contraindications. Participants complete a urine drug screen (Alere Multi-drug Screen Test Panel; DOA-3104) before the scan. Scans are conducted on a 3T Siemens Prisma scanner using a 20-channel head coil. Collected scans include a T1, 3D-pseudo continuous arterial spin labeling, blood oxygenation level–dependent scan for resting state functional connectivity, diffusion basis spectrum imaging, and fluid-attenuated inversion recovery. For anatomical and pathological analysis, high-resolution magnetization-prepared rapid acquisition and T2 scans are acquired. Arterial spin labeling is acquired using a standard sequence (repetition time =3500 seconds, echo time =9 milliseconds, 90° flip angle, voxel size=3.4×3.4×5 voxels, 64×64 acquisition matrix, 22 axial slices with a 1-mm gap). We use pipelines developed by the Washington University in St. Louis Central Neuroimaging Data Archive and FreeSurfer software (version 5.3) to process the imaging data. Brain volumes and CBF are the primary outcome variables generated from MRI scan data. Additional data acquired from the performed sequences, including blood oxygenation level–dependent scan and diffusion basis spectrum imaging data, are considered in secondary analyses.

##### NP Testing

Participants complete the questionnaires and NP testing after the MRI. Web-based questionnaires assessing mental health, substance use, sleep quality, and subjective memory performance are first administered using an iPad (iPad Air 2, model MH182LL/A; Table 1). Following the questionnaires, the NP testing begins with allowances for breaks when needed. Most tests are administered in paper and pencil format, with some tests administered on the iPad. Questionnaires and NP testing take 2-3 hours to complete (Textbox 1).

**Table 1:** Questionnaires administered at baseline and end point visits.

Domain and questionnaire			Function
**Substance use**			
	KMSK^a^-Lifetime [33]	Quantify lifetime heaviest use of alcohol, tobacco, marijuana, cocaine, heroin, and opiates	
	KMSK-30 Day [33]	Quantify use of alcohol, tobacco, marijuana, cocaine, heroin, Rx benzodiazepines, illicit benzodiazepines, Rx opiates, illicit opiates, illicit barbiturates, amphetamines, and methamphetamines in past 30 days	
	Fagerstrom Test for Nicotine Dependence [34]	Nicotine dependence	
**Sleep**			
	Pittsburgh Sleep Quality Index [35]	Measures quality and patterns of sleep	
**Activities of daily living**			
	Patient Assessment of Own Functioning [36]	Measures perceived daily functioning impairments	
**Activity**			
	International Physical Assessment Questionnaire [37]	Measure of physical activity	
	Patient-Centered Assessment and Counseling for Exercise [38]	Assess physical awareness	
**Mood**			
	Beck Depression Inventory-II [39]	Measures characteristic attitudes and symptoms of depression	
	Centers for Epidemiologic Studies-Depression Scale [40]	Measures self-reported symptoms associated with depression	
**Memory**			
	Prospective Retrospective Memory Questionnaire [41]	Self-report measures of prospective and retrospective memory slips in everyday life.	

^a^KMSK: Kreek-McHugh-Schluger-Kellogg scale.

Neuropsychological (NP) test battery. All tests are completed at baseline and end point NP visits. Tests denoted with a “*” are also completed at the midpoint NP visit.

**Learning and memory domain**

- Hopkins Verbal Learning Test–Revised* [42]
- Brief Visuospatial Learning Test Revised* [43]

**Prospective and retrospective memory domain**

- Virtual week program [44]

**Executive function domain**

- Action Fluency* [45]
- Trail-Making Test B* [46]
- Letter Number Sequencing* [47]
- Paced Auditory Serial Addition* [48]
- Color Word Inference Test–Stimuli 3* [49]
- Digit Span-Backwards [47]
- Medication Management Task–Revised [50]
- Wisconsin Card Sorting Task [51]
- Rey-Osterrieth Complex Figure–Copy* [52]

**Psychomotor or processing speed domain**

- Trail Making Test-A* [46]
- WAIS-III Symbol Search [47]
- Digit Span* [47]
- Digit-Symbol Substitution Test* [47]

**Language domain**

- FAS fluency* [53]
- Animal fluency [54]

**Motor and fine motor domain**

- Grooved Pegboard (dominant and nondominant hand)* [55]
- Finger tapping*
- Timed Gait
- Grip strength

**Premorbid IQ**

- Wide range achievement test 3–reading and recognition [56]

#### Study Arm Randomization

Group randomization is performed after all baseline appointments are completed and the participant is determined to be safe to exercise. A randomization program is employed by the study senior statistician to assign group membership with a 2:1 ratio in EXS and SIS arms, respectively. All study staff directly involved with participant testing are blinded to the randomization; only the personal trainer is aware of the study arm designation. All participants are instructed not to disclose their randomization to study staff.

### Interventions

#### Overview

Each participant is familiarized with the gym facility where the intervention takes place. At the first session, participants are given a Polar chest HR monitor and then instructed to complete a 5-minute warm-up (options include walking on the indoor track, stationary cycling, elliptical, or treadmill). After warm-up, participants complete a 6-minute walk test [57] as an assessment of cardiovascular fitness. Distance walked in meters during the 6-minute period is recorded. The 6-minute walk test is repeated several times throughout the course of the study intervention for all participants, at intervention visits 20, 40, 60, and at the final visit.

For all subsequent intervention visits, HR is recorded through use of the Polar chest HR monitor, and the 5-minute warm-up is completed before beginning the EXS or SIS session.

Participants are scheduled for 3 intervention visits per week, with all visits supervised by the personal trainer. A participant’s intervention is complete after 26 weeks have elapsed. Adherence is defined as the participant completing ≥80% of the expected 78 intervention visits.

#### EXS Intervention

After the 5-minute warm-up, the participant has the option to start either the cardiorespiratory training portion or resistance training portion first, followed by the other for the second half of the daily session. Resistance training covers a full-body workout including 3 lower body and 4 upper body areas, with exercises including leg press, knee extension, knee flexion, seated row, chest press, latissimus dorsi pulldown (lat pulldown), and triceps extension. A combination of guided motion machines and free weights is used.

At the second gym session, a 1-repetition maximum (1RM; measured in pounds) is assessed for each of the major muscle group stations (leg press, lat pulldown, seated row, and chest press) to establish peak muscular strength. The rate of perceived exertion scale (1-10) is used to measure intensity. The 1RM is reevaluated after each month.

During the course of the EXS intervention, the trainer begins the resistance training program with weight for each exercise set to 60% of each 1RM. Three sets of 8-10 repetitions are completed for each exercise for the first 3 months. The trainer increases the weights to 70% of the reevaluated 1RMs for the final 3 months. A rest period of 2 seconds is allowed between each repetition, 1-2 minutes of rest is allowed between each set, and 2-4 minutes of rest between each exercise station or machine.

Cardiorespiratory training includes brisk walking on the indoor track, stationary cycling, treadmill, and elliptical machines. Participants may use a combination of different cardiorespiratory machines to reach their target duration and HR. Target HR begins at 50% of the heart rate reserve (HRR) as assessed during the initial cardiorespiratory testing. Throughout the study, the target HR increases to 85% of the participants’ HRR. Duration is progressively increased from 10 to 40 minutes of continuous exercise as the participant acclimates to the exercise program. Intensity is increased as exercise yields a lower HR than previously observed or by 5%-10% per month. After each session, the participant cools down to bring their HR to a comfortable level that would allow conversation. The trainer then demonstrates a stretching routine that covers the major muscle groups of the body (quadriceps, hamstrings, hips or glutes, chest, back, shoulders, and triceps).

#### SIS Intervention

The SIS group serves as a control group against which to gauge the effects of aerobic and resistance training on brain integrity. Participants in this group follow the same intervention schedule and timeline as the EXS group. SIS participants are supervised by the same personal trainer and receive the same amount of attention and class interaction as participants in the EXS program. This group receives instructions on stretching, range of motion (RoM), limbering, and toning, but intensity is less than that achieved in the EXS group. Activities will focus on flexibility enhancement. As the participant’s level of flexibility increases, stretches with increasing levels of difficulty are incorporated into the program.

For the stretching intervention, the participant begins with the 5-minute warm-up. During the second session, the trainer conducts RoM testing for hip flexion, shoulder flexion, ankle dorsi, and plantar flexion on both sides of the body using a goniometer. The RoM is reevaluated after each month.

During each SIS visit, the trainer demonstrates and provides a list of stretches that may include but are not limited to the following muscle groups: hamstrings, gluteals, lower back, upper back, chest, calves, shoulders, triceps, and arm and wrists. Participants complete 10-12 stretches with 30-60 second intervals for each stretch. These stretches are repeated 2-3 times on each side of the body during each session. HR is maintained between the established resting HR and 50% of the HRR.

### Midpoint (3-Month) Visit

Once the participant has completed 3 months (36-40 gym visits), a midpoint visit is scheduled. At the midpoint visit, an abbreviated NP battery is administered (see Textbox 1). This battery includes tests that were administered during the baseline NP testing, with alternative versions of NP tests used when available. Additionally, the participant is again provided an Actigraph to be worn for 1 week in order to assess daily activity during participation in the study.

### End Point (6-Month) Visit

Once the intervention portion (78 sessions) is completed, the end point (6-month) visits are scheduled. End point visits are repeats of the baseline visits 1 and 2, with the cardiovascular testing visit occurring first. The DXA scan and VO_2 max_ test are completed, fasting blood samples are collected, and the participant is given an Actigraph to wear on the nondominant wrist for 1 week. After 1 week, the participant returns for the follow-up MRI and NP testing visit. Upon completion of the study, participants are given a 6-month membership to a local fitness facility.

Midpoint and end point visits are completed for all participants, regardless of assigned intervention.

### Secondary End points

This study aims to also identify key implementation factors needed for scale-up of an EXS program in older people living with HIV from a single site to HIV clinics at multiple institutions. In order to gain a sound understanding of contextual factors that affect implementation of an EXS program for older, sedentary people living with HIV, we are conducting interviews and focus groups with staff and participants at several time periods during the intervention. This allows us to (1) tailor aspects of the program to coincide with reported barriers to implementation, (2) develop strategies to enhance adherence, and (3) make recommendations for future intervention strategies based on modifiable psychosocial characteristics of people living with HIV as they progress through the intervention.

### Planned Recruitment and Power Analyses

The recruitment goal for this study is 150 older participants living with HIV, with 100 randomized to the EXS intervention and 50 participants randomized to the SIS control intervention. With this sample size, we calculated 80% power to detect an effect size (Cohen *d*=0.49) in the primary analyses (aim 1 hypotheses *a* and *b*; aim 2 hypothesis *a*). Anticipating a 20% dropout rate at 26 weeks (final EXS n=80; SIS n=40), we would have 80% power to detect an effect size (Cohen *d*=0.55). For aim 2 hypothesis *b*, and anticipating a 20% dropout rate, we would have 80% power to detect a predictor explaining *R*^2^=0.10 or 10% of the variation in the outcome.

### Statistical Analyses

For aim 1, planned statistical analyses include *t* tests examining change in cognition (domain and global cognition *z* scores), cardiorespiratory fitness, physical function, and questionnaire data from the baseline to end point visits between the EXS and SIS groups. This analysis uses all randomized participants under the intent-to-treat paradigm. Additionally, linear models are used to determine the mediating effect of the cardiorespiratory fitness and physical function markers on the change from baseline in domain and global cognition *z* scores by including these markers, together with group assignment, as predictors in the model.

For aim 2, similar analyses are conducted to those as described for aim 1, with brain volumetrics and CBF values used in place of cognitive performance. Brain volumes are adjusted for intracranial volume. Linear regression analyses are conducted to examine relationships between changes in brain volumetrics or CBF and changes in cognitive performance from baseline to end point visits.

For the exploratory hypothesis, linear regression models are used to examine relationships between changes in inflammatory or metabolic markers and changes in brain volumetrics, CBF, or cognitive performance. Group assignment is included as a predictor in the model.

### Ethics Approval

Before completing any study procedures, all participants provided written informed consent that was approved by the Institutional Review Board at Washington University in St. Louis (IRB #201508002). All study procedures were in accordance with the ethical standards described by the Helsinki Declaration. Study participants are assigned an identification number, allowing all stored records to be deidentified. Deidentified data are kept in a locked file room or stored in a password-protected database on a password-protected computer. All participants are monetarily compensated for their participation, prorated to each study visit or gym visit completed, up to US $815. Participants can withdraw from the study at any point.

## Results

**Figure 1:**
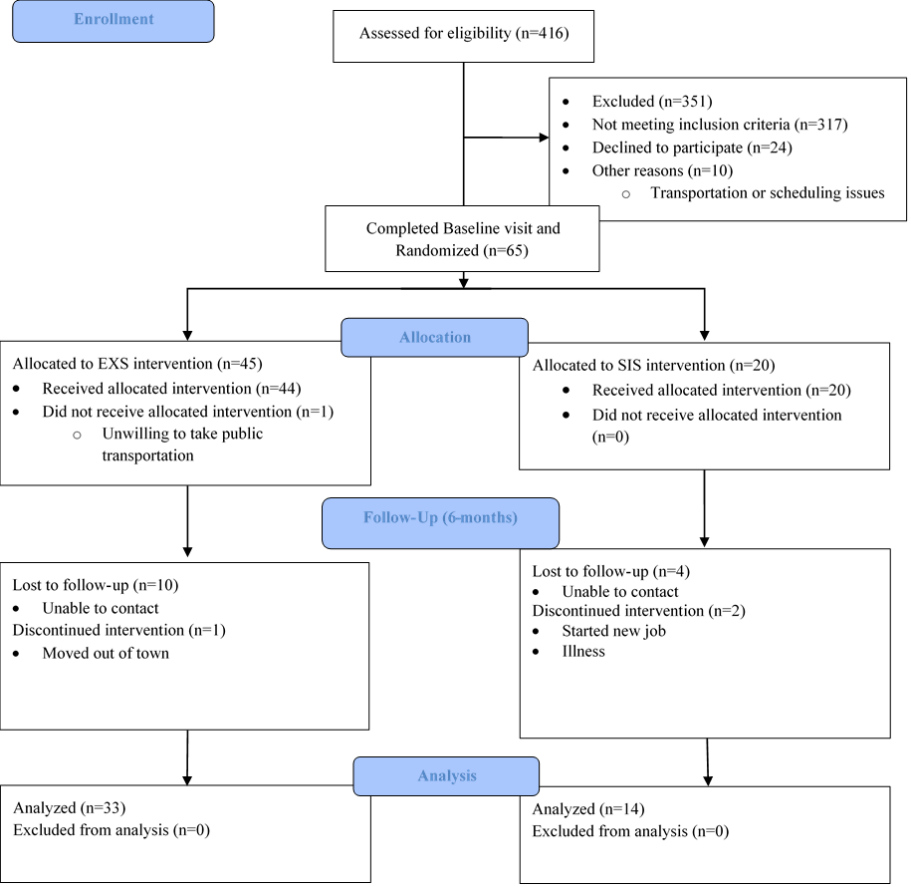
CONSORT (Consolidated Standards of Reporting Trials) flow diagram of study enrollment. EXS: aerobic and resistance exercise; SIS: stretching.

Recruitment and data collection are complete as of December 2020 (Figure 1). Recruitment and data collection were paused between March 2020 and September 2020 due to the COVID-19 pandemic and again paused in November 2020 due to increasing COVID-19 cases. Consequently, the decision was made to terminate the recruitment phase due to limited time for any newly enrolled participants to complete the 6-month intervention. Baseline study participant demographic and clinical characteristics are provided in Table 2. A total of 65 individuals were enrolled and randomized at a baseline visit, with 47 completing the study. As of July 2022, the study remains open for data processing and analyses. Data processing, cleaning, and organization are complete as of December 2021. Data analysis began in January 2022, with publication of study results for primary aims 1 and 2 expected by early 2023.

**Table 2:** Baseline demographic and clinical characteristics by intervention group.

	EXS^a^ group (n=45)	SIS^b^ group (n=20)	*P* value
Age (years), mean (SD)	56.5 (5.7)	56.5 (5.9)	.99
Education (years), mean (SD)	12.9 (2.4)	13.9 (2.2)	.14
Sex (male), n (%)	32 (71)	18 (90)	.10
BMI, mean (SD)	30.5 (7)	28.9 (5.7)	.39
Ethnicity (African American), n (%)	29 (64)	14 (70)	.81
Recent CD4 T-cell count (cells/mm^3^), median (IQR)	581 (474-817)	561 (362-771)	.47
CD4 nadir T-cell count (cells/mm^3^), median (IQR)	195 (22-312)	47 (25-358)	.84
CD8 T-cell count (cells/mm^3^), median (IQR)	809 (489-1079)	689 (577-1035)	.50
Duration of HIV infection (months), mean (SD)	225.4 (117.6)	259 (94.3)	.27

^a^EXS: aerobic and resistance exercise.

^b^SIS: social-interaction stretching.

## Discussion

Exercise interventions have demonstrated beneficial effects on physical fitness and brain integrity in middle-aged to older adults without HIV infection [4-7]. However, few studies have examined these relationships in people living with HIV. Adjunctive therapies designed to improve brain integrity are needed as the population of people living with HIV over the age of 50 years continues to grow and experience cognitive impairment and decline despite cART and well-controlled viremia. This study was designed to examine the effects of a 6-month exercise intervention on cardiometabolic risk, cardiorespiratory and physical fitness, cognitive performance, and neuroimaging metrics of brain integrity in a sample of sedentary people living with HIV aged ≥40 years on stable cART and who are virologically well controlled.

This study has several important strengths that underlie its potential contributions to the field of therapeutic interventions for older people living with HIV. First, this study recruits a sizeable sample of sedentary people living with HIV, primarily over the age of 50 years, to complete an exercise intervention or an attention control program involving stretching. To date, the few interventional studies examining effects of an exercise program in people living with HIV have included a relatively small number of individuals, limiting the generalizability of the results, and have primarily been conducted in younger (age ≤40 years) individuals. However, people living with HIV aged >50 years are estimated to make up over half of all individuals currently living with HIV in the United States [58] and are at a higher risk for cognitive impairment compared to younger individuals [59,60]. Additionally, this group of people living with HIV is well characterized in terms of physical health, blood inflammatory markers, cognitive ability, and brain imaging, allowing for in-depth analyses examining the effects of the EXS intervention on these outcomes. The 6-month duration of the intervention with midpoint data collection also allows for the potential capture of both acute and long-term effects of exercise on the brain in people living with HIV. Finally, the inclusion of a focus group will help inform future exercise programs for people living with HIV by assessing the feasibility of such programs barriers and facilitators for enrollment and retention, as well as predictors of successful adherence to the program.

This study design does have inherent challenges. Recruitment and retention are challenges for any prospective study requiring lifestyle changes for a long duration. Our inclusion and exclusion criteria require a participant to be healthy enough to safely participate in a 6-month supervised exercise program, limiting its generalizability; however, this requirement selects a population who are potentially the most likely to tolerate and complete the study intervention and therefore benefit from participation. Finally, the nature of the study may also introduce a recruitment bias into the sample, as people living with HIV who are willing and able to travel to the study site 3 times a week for 6 months during normal working hours may have baseline differences in socioeconomic status, physical health, and cognitive ability than people living with HIV who are not able to meet these requirements. However, by focusing recruitment on a diverse sample (eg, sex, race or ethnicity, education level, and cognitive ability) and by using group interviews to systematically assess these obstacles, we hope to broaden the generalizability of this intervention to a larger proportion of the population living with HIV. Results gained from this study will help inform clinicians as to possible benefits gained from prescribing an exercise program to middle-aged to older people living with HIV who may be experiencing cognitive issues, as well as help advise the development and implementation of future exercise programs for people living with HIV.

## Abbreviations

1RM: 1-repetition maximum
BP: blood pressure
cART: combination antiretroviral therapy
CBF: cerebral blood flow
DXA: dual energy x-ray absorptiometry
EXS: aerobic and resistance exercise
HR: heart rate
HRR: heart rate reserve
MRI: magnetic resonance imaging
NP: neuropsychological
RoM: range of motion
SIS: social-interaction stretching
VO2 max: VO2 maximum

## Data Availability

The data sets generated and analyzed during this study are available from the corresponding author on reasonable request.
